# Expectation Violation in Political Decision Making: A Psychological Case Study

**DOI:** 10.3389/fpsyg.2017.01761

**Published:** 2017-10-16

**Authors:** Michael Öllinger, Karin Meissner, Albrecht von Müller, Carlos Collado Seidel

**Affiliations:** ^1^Parmenides Foundation, Pullach, Germany; ^2^Department of Psychology, Ludwig Maximilian University of Munich, Munich, Germany; ^3^Institute of Medical Psychology, Ludwig Maximilian University of Munich, Munich, Germany; ^4^Division Integrative Health Promotion, University of Applied Sciences Coburg, Coburg, Germany; ^5^Department of Philosophy, Ludwig Maximilian University of Munich, Munich, Germany; ^6^Department for Modern and Contemporary History, Phillips University of Marburg, Marburg, Germany

**Keywords:** problem-solving, expectancy, expectation violation, political-decision-making, mental set

## Abstract

Since the early Gestaltists there has been a strong interest in the question of how problem solvers get stuck in a mental impasse. A key idea is that the repeated activation of a successful strategy from the past results in a mental set (‘Einstellung’) which determines and constrains the option space to solve a problem. We propose that this phenomenon, which mostly was tested by fairly restricted experiments in the lab, could also be applied to more complex problem constellations and naturalistic decision making. We aim at scrutinizing and reconstructing how a mental set determines the misinterpretation of facts in the field of political decision making and leads in consequence to wrong expectations and an ill-defined problem representation. We will exemplify this psychological mechanism considering a historical example, namely the unexpected stabilization of the Franco regime at the end of World War II and its survival thereafter. A specific focus will be drawn to the significant observation that erroneous expectations were taken as the basis for decisions. This is congruent with the notion that in case of discrepancy between preconceived notions and new information, the former prevails over the new findings. Based on these findings, we suggest a theoretical model for expectation violation in political decision making and develop novel approaches for cognitive empirical research on the mechanisms of expectation violation and its maintenance in political decision making processes.

## Fixation, Mental Set, and Expectation

In the year 1935, 4 years before the Second World War started, Karl Duncker published his seminal book “Zur Psychologie produktiven Denkens” (Translated to English in 1945: “On problem solving”) (Duncker, 1935, 1945). In his book Duncker founded a theory which has been widely influencing the research of insight problem solving until today. Insight problems are characterized by having either no obvious or just step-wise solutions, but they have a sudden, unexpected, and unintended character. Usually, the solution requires a re-structuring of the problem elements or the assumptions that were imposed on the problem (Metcalfe and Wiebe, 1987; Ohlsson, 1992, 2011; Wegner, 2002; Öllinger and Knoblich, 2009). Duncker was fascinated by the question, what factors block the problem solving process and make people blind to insightful solutions.

In his famous candle problem he asked participants to fix a candle on the wall – given a matchbox, a box full of tacks and a candle. The problem proved to be extremely difficult. The solution proceeds as follows: empty the box and fix it with the tacks to the wall, then light the candle, put wax on the matchbox and glue the candle onto the box. Duncker argued the problem was difficult because participants fixated on the usual functions of the given objects. In this case, the given box needs to be used as a container, not as a ledge. This example demonstrates that prior knowledge imposes constraints on the utilization of objects. Similarly, Maier showed a few years earlier (Maier, 1931) that participants had problems using an object (e.g., a pair of pliers) as a weight for a pendulum.

**FIGURE 1:**
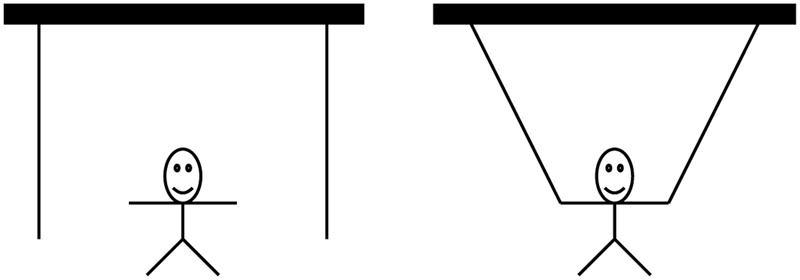
Maier’s two-string problem. Left: Initial state. Right: Goal state.

This is part of the solution to the two-string-problem (see **Figure 1**). Participants were asked to tie two strings together which hung from the ceiling. The distance between the strings was too far apart to catch hold of both at once. The “insightful” solution to this problem is to use the pair of pliers as weight on one string and to swing it like a pendulum. Thus, both ends of the strings can be reached. Participants fixated on the usual function of the pair of pliers. Therefore, they had difficulties to use the objects’ weight properly.

Luchins (1942) demonstrated that fixation was not only restricted to object properties, but could also be induced by the repeated activation of the same successful solution procedure. Luchins asked participants to solve various water-jug-problems. The objective was to fill a certain amount of water into one of three jars with different capacities. The capacities of the empty jars in the first experiment were: A (21 units), B (127), and C (3). The goal was to attain exactly 100 units by pouring the water from one to another. The solution of the problem is to fill water into B (127), then pour water from B (127) to A (21) = B (106) and finally twice from B (106) to C (2 × 3) = B (100). Luchins provided a sequence of analogous problems which could always be solved with exactly the same sequence. After a sequence of similar problems, a test problem was presented, which could be solved either by the usual sequence or in a much easier way [e.g., A(23), B(49), and C(3) goal state was 20]. The easy solution is to pour water from A to C. Almost two thirds of the participants who were trained with the difficult strategy were blind to the easy solution. They were caught in a mental set. Luchins also showed that even in the case of a problem which obviously could not be solved by the learned procedure, participants tried the usual strategy and re-applied it subsequently when a new problem was presented. It seemed fairly difficult to overcome the mental set and the associated expectations.

Lovett and Anderson (1996) proposed a computational model for mental set. It demonstrated that by increasing the weight of a procedure after each successful attempt the probability of its application will increase.

Öllinger et al. (2008) combined the concept of mental set with the domain of insight problem solving. They demonstrated that telling participants an insightful solution to a problem and repeating the same solution strategy several times inhibited the likelihood that participants applied a standard solution to problems – even if they did not require any insightful problem solving. As a result, this means insight blocked well-known prior knowledge strategies.

To conclude, we propose that fixation and mental set induce rigid behavior – firstly by exploiting prior knowledge and secondly by procedural and working memory activation. It seems conceivable to assume that both mechanisms influence the problem solvers’ expectation. Fixation constrains the expectation about the utilization of an object. Mental set creates an expectation about the most promising and efficient strategy. By failures, expectations are violated and participants get stuck in an impasse or reluctantly repeat the wrong solution approach (Smith and Blankenship, 1989, 1991; Fedor et al., 2015; Öllinger et al., 2016).

Carnevale and Probst (1998) investigated the question what kind of mental sets were introduced by either social conflicts or social cooperation. In a first experimental group, they induced a conflict by giving the information that others will compete in a negotiation situation. In the second group, participants were informed that others want to cooperate with them. After mental sets were induced, the participants were asked to individually solve Duncker’s candle problem. It turned out that participants in the conflict situation were significantly less likely to find the creative solution (empty the box and use it as a platform) than in the cooperation set. The authors emphasized that considering a situation as a conflict “promotes a freezing of knowledge” (p. 1301).

Bar-Tal et al. (1989) suggested that changing expectations could help to resolve a conflict mental set and convey it to a cooperation set. The authors stated that a social conflict is a cognitive schema. The schema is associated with knowledge and implications emanated by core beliefs.

Kruglanski (2013) postulated two processes. The first is the generation phase, which generates cognitive content. The second is the cognition validation phase, where a degree of confidence is mapped to the generated content. The first phase is crucial for potential mental set in our framework, since it selectively sets its focus on selected and biased pieces of information. In the second phase, persons test the generated information with stored evidence, whether the information is logically consistent or not.

Bar-Tal et al. (1989) detailed on potential processes which influence the generation process. They argued that parties have a need for closure, which means to stick to certain beliefs or maintain a particular belief as true and reject contrary ideas or alternative perspectives. To resolve a conflict, it is necessary to alternate the cognitive schema. The expectation that a conflict will continue will not change the accessibility of the conflict schema.

For our line of argumentation this means that the violation of expectations will activate the conflict schema. The repeated activation of this mismatch will strengthen the conflict and at the same time strengthen the core believe that there is only one solution to the given problem – the person gets stuck in a mental impasse.

To sum up, there is evidence that decision making processes are negatively influenced by the repeated activation of an apparently successful solution strategy or apparently related information. Furthermore, successful strategies appear to increase the likelihood for a premature closure making blind for alternative solution approaches. Regarding political decision making, this may imply that political actors can find themselves in a conflict mode which prevents creative thinking necessary to solve a difficult problem.

In the following, we aim at contextualizing historically documented facts based on a comprehensive archival research (see Collado Seidel, 2016) with the purpose of demonstrating the relevance of mental sets in the domain of political decision making. We will exemplify this psychological mechanism considering the unexpected stabilization of the Franco regime at the end of World War II and after. Based on cognitive models, we aim to explain the persistence of a contradiction between expectations on the one hand and the rational perception of given facts pointing in the opposite direction on the other hand. A specific focus will be drawn to the significant observation that erroneous expectations were taken as the basis for decisions, showing that in case of discrepancy between preconceived notions and new information the former prevail over the new findings.

## Franco and the Expected Post-War Order: Reconstruction of Expectation Violation in a Historical Context

The Franco regime was considered an intrinsic part of the fascist European order during World War II (see Bowen, 2000; Collado Seidel, 2001, 2005, 2012). This circumstance was clearly perceived by the British and Americans. As a striking example, in 1940 the British ambassador to Madrid, Sir Samuel Hoare, wrote to a member of the Cabinet: “I have never seen so complete a control of the means of communication, press, propaganda, aviation, etc., as the Germans have here. Indeed, I go so far as to say that the Embassy and I are only existing here on German sufferance” (Hoare, 1946, p. 32).

Therefore, American and especially British political observers and decision makers, who had the leading role in the definition of Allied politics toward Spain, expected that with the crushing of German Nazism and Italian fascism, the Spanish dictator would inevitably fall as well. A radical change, hopefully by democratic forces, was expected and the conviction persisted that this outcome was merely a matter of time and the “problem Franco” would solve itself.

This conviction was consequently expressed by diplomats and politicians involved and was shared at the top level of the British and American governments as well (Hull et al., 1943; Hollis, 1944). It can be illustrated with one of Hoare’s vivid appraisals, dated June 1943: “The Spanish tide is, in fact, running in our favor and, this being so, I should let it take its own course, and not attempt to force its pace. [...] It will, in my considered view, collapse all the sooner if we leave it to the Spaniards themselves to give it the *coup de grâce*. [...] The evidences available in Spain go to show that it will be a monarchist restoration, and that the restoration will be attempted between now and the end of the year” (Hoare, 1943). Oliver Harvey, the Principal Private Secretary to the British Foreign Secretary, put it straight shortly thereafter with his remark: “Damn Franco! We’ll have him off his perch before we are done” (Harvey, 1943).

Remarkably, this wrong expectation was maintained despite the perception that Franco was even strengthening his power within Spain. Psychologically, one may assume that the responsible decision makers suffered from a pronounced mental set which drove their judgment.

## Expectation: The Franco Regime Will Not Survive World Word II

The expectation of the Allied that the Franco Regime would not survive World War II was based on three mutually related assumptions:

(1) If the fascist reference system (Axis Powers) collapses, Spain will be destabilized.

(2) The opposition in Spain will exploit the weakness of the Franco regime and will establish a new regime along democratic structures; otherwise, revolutionary events will force the issue.

(3) The Spaniards are eager to get rid of Franco.

In anticipation of the results: All of these expectations were proven wrong and in the end the Franco regime proved stable and kept control until 1977, when a new democratic constitution was worked out and the first free elections were held in Spain.

In the last stages of World War II, however, the British and American governments started from the premise that Franco’s Spain would not survive the collapse of the Axis Powers. Furthermore, observers – such as the British ambassador – were convinced that the dissatisfaction with the regime was growing continuously in all sectors of Spanish society. Ambassador Hoare professed moreover his belief that the vast majority of Spaniards, even the working class, favored the restoration of the Monarchy (Hoare and Greville, 1942; Hoare, 1944).

The American ambassador to Spain, Carlton Hayes, basically shared this view, though he, as the Americans in general, favored the establishment of a Republic. Furthermore, though he had serious doubts whether the Spaniards really preferred the Monarchy being restored, he firmly shared the conviction that the vast majority of Spaniards detested the Falange and that Franco would leave power either voluntarily or forcibly, giving way to a new regime (Hayes, 1944). In case of Franco’s refusal to permit a political transition or to introduce radical changes in the structure of his regime, the political observers expressed their conviction that the dictator would be forced to leave power, as ambassador Hayes put it in May 1943: “If Franco gets rid of the Falange in time (which I imagine he won’t), he may be able to lead an evolution toward a more liberal government and to retain a place in it. Otherwise, he will be forcefully ousted along with the Falange” (Hayes, 1943).

The expectation of a sudden breakdown of the Franco regime became even more intense and bordered on certainty after the dismissal of the fascist dictator Mussolini in July 1943: Observers like Alan Hillgarth, the key person of British intelligence services in Spain, or George Kennan, special envoy of the US-State Department, were persuaded that the Italian events would shortly find a repetition in Spain (Hillgarth, 1943; Kennan, 1943).

In sum, there existed no doubts that Franco’s end was at hand. The British and Americans maintained this conviction as shown exemplarily in a dispatch from the US-ambassador, dated September 1944: “The régime, as it is, can hardly survive the final outcome of the war. In a Europe, and a world, then turning more and more ‘leftward,’ Spain could not remain apart and insulated from such a universal current” (Hayes, 1944).

Contrary to these expectations, however, neither the destitution of Mussolini in July 1943 nor the landings of the Allied Forces in Normandy in June 1944, and not even the collapse of Nazism in May 1945 shook the Spanish regime. Nevertheless, the Western Allies perseverated on their view, as a British diplomat put it in October 1945, though in a somehow exasperated way in the face of the past experiences: “Franco’s down-fall is only a matter of time, whether weeks, or months or years” (Garran, 1945b). Realizing that the expected changes did not occur, the political analysts seemed stunned and helpless and got stuck in an impasse: “There is a Spanish political reality entirely apart from the general European situation, i.e., Franco needs not fall with Hitler though undoubtedly Franco will fall if he does not change in time” (Bonsal, 1944).

## Perceptions Running Contrary to the Expectations

This conviction professed by the British and Americans is especially surprising due to the circumstance that the same political observers perceived the weakness and disunity of the oppositional forces to compel a change and on the contrary, the political system showed no signs of weakening. The monarchists as the most promising group just launched weak attempts by presenting writings in which they urged in favor of the restoration of the Monarchy. In addition, they were profoundly divided in view of the pursued aims after the downfall of Franco. This was understood very clearly: “The strong sectionalism and individualism within Spain appear to foster sectional and individual political aims which transcend national political aims, with consequent lack of any unifying national program or organization for the opposition as a whole or even for any considerable part of it” (Hayes, 1944). The same basic problem was seen in the case of the republican movement, whose only common factor was to overthrow the existing regime. Even the communist guerrilla, as the best organized opposition group, showed itself unable to force a shift and to provoke a general uprising, as demonstrated by the failure of the incursion of some thousand guerrilla fighters in the Pyrenees in autumn 1944.

In view of these developments, as early as February 1944, the British general staff reached the conclusion that no existing political or military group was in a position to oust Franco (Joint Planning Staff, 1944). Half a year later, a British political analyst summarized the situation in Spain in the same way by stating: “Contrary to normal expectations General Franco’s position is undoubtedly stronger in Spain today than it had been at any time during the past few years” (Roberts, 1944). Even after the Hitler regime collapsed, Franco showed no tendency to change his politics. He had apparently even consolidated his position, as assessed by a British diplomat: “Franco is at present more firmly established in power than ever, in spite of the defeat of his fellow dictators” (Garran, 1945a).

Besides the observation that the opposition was deeply divided, British and American analysts realized quite early the presumably most ponderous reason for the hesitant attitude of the Monarchists as the most promising opposition group: the fears of provoking a communist revolution and the return of uncontrolled violence and chaos in case of a sudden political change and in particular by destabilizing the system in trying to overthrow Franco forcibly. The profiteers of the outcome of the Spanish Civil War did not want to endanger their own privileged economic and social position for the sake of bringing back the King. In the end, they felt quite comfortable with the prevailing situation. Thus, Hoare remarked with an amazed undertone he had “never known so many professed monarchists who didn’t really want a king” (quoted in Hayes, 1946, p. 269).

Furthermore, the Allied perceived that Franco not only presented himself as the sole person able to prevent the country from plunging into chaos and anarchy. As stated by British diplomats, in view of the ostracism practiced by the British and American governments the dictator managed to present the criticism against his regime and his person as an attack on the Spanish nation, achieving broad public support (see Portero, 1989, p. 217; Collado Seidel, 2015, pp. 178f.).

## Decision Makers in Conflict

The fear of provoking a renewed civil war hindered not only the Monarchists in their attempts to overthrow the Franco regime. The Allied, and especially the British policy makers as spokesmen of the common politics toward Spain, were as well discouraged from pursuing a more straightforward policy accompanied by the imposition of effective economic sanctions such as an oil-embargo for the same reason. Churchill put it straight by remarking toward a more receptive Foreign Secretary: “What you are proposing to do is little less than stirring up a revolution in Spain. You begin with oil: you will quickly end in blood” (Churchill, 1944b).

But it was not just the memory of the atrocious civil war, which hindered the enforcement of a rather stiff attitude toward Franco. Above all, Churchill had in mind the Soviet Union and its alleged interests in provoking a revolution and a communist takeover on the Iberian Peninsula in mind. The chaotic and troublesome situation in Europe after the war led Churchill to avoid the embarkment toward new political adventures (Churchill, 1944a).

## Wrong Assumptions and Misperceptions

Our historical analysis allows us to assess the information disregarded by the Allied political decision makers, which prevented them to build up a proper representation of the problem. We reconstruct the facts that were available but were not integrated in a proper problem representation.

First, the assumption that the successful elimination of Hitler’s Nazism and Mussolini’s fascism would result in the downfall of the Franco regime, due to the consideration that it constituted an intrinsic part of the fascist European order, was the main misconception and led to dramatic misjudgements. Not least, the political actors were influenced by the events which led to the destitution of Mussolini. This resulted in a “sit and wait” attitude in the expectation that the problem solves itself.

Second, it was overestimated that the Spanish monarchical and republican opposition would exploit the doom of fascism bundling their forces with the aim of overturning the regime. On the contrary, the opposition was weak and disunited.

Third, while it can certainly be assessed that the dictator and the dictatorship were by no means popular, it proved wrong that the Spaniards were eager to get rid of the regime. This was not just the case in view of the monarchical opposition, which preferred not to run risks, which could endanger their own privileged position, but also for the vast majority of the population who was afraid of chaos and a renewed civil war, as shown by the lack of support the activities of the guerrilla received.

Furthermore, the British and American decision makers were trapped in a conflict mode regarding the putative reactions of the Soviet Union as a competing player: the risk of a revolutionary insurrection and a renewed civil war, which could lead to negative consequences for themselves, resulted in a narrow-minded assessment of the situation that impeded considering alternative procedures as postulated by Carnevale and Probst (1998).

To sum up: British and American observers and decision makers gained and discussed the information which proved the basic assumption of an imminent end of the Franco dictatorship wrong. Despite seriously analyzing the information, they adhered firmly to their original considerations. The search for alternatives may further have been hindered by a conflict mode resulting from the perception of the Soviet Union as a competing player. As a result, the political key players were stuck in an impasse, which they were unable to solve, and which contributed, in a historical perspective and against all expectations, to the outcome of the survival of the Franco regime after World War II until 1977.

## Analogies Between Impasse in Problem Solving and Historical Deadlocks

This article follows the credo of the famous Gestaltist and social psychologist Kurt Lewin, stating that “there is nothing as practical as a good theory” (Tolman, 1996, p. 31). We now aim at bridging the gap between basic research on problem solving and the complex and applied historical situation presented above.

Expectations in problem solving, as described above, are driven either by prior knowledge or the repeated activation of a successful solution procedure (Lovett and Anderson, 1996; Ohlsson, 2011). Recently, Öllinger et al. (2016) demonstrated that even the repeated activation of an inappropriate solution strategy led to fixation on this strategy, although participants received consequent negative feedback.

**FIGURE 2:**
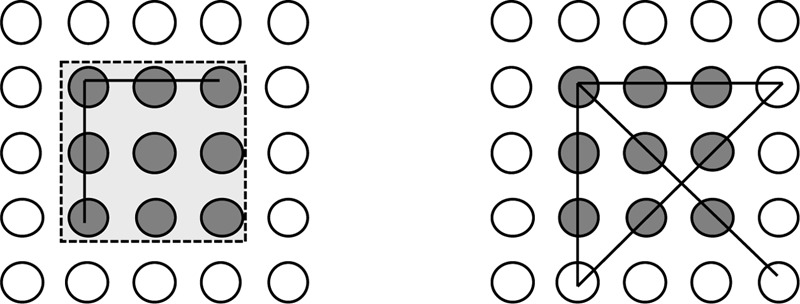
Left: The constrained Nine-Dot problem. Right: A possible solution of the problem. In the beginning the Nine-Dot problem seems very simple and straightforward. Initially, problem solvers expect that the given nine dots need to be connected by dot-to-dot connecting lines and the self-imposed assumption that lines should stay within the boundaries of the given nine dots.

The reason might be that within the ill-defined search space there were no other possibilities. This could nicely be demonstrated by the Nine-Dot problem (see **Figure 2**). The task is to connect the given nine dots by four straight and connected lines. It is not allowed to lift the pencil or to retrace a line (Maier, 1930; Scheerer, 1963). The problem proved to be extremely difficult and reluctant to clues or hints (Lung and Dominowski, 1985; Chronicle et al., 2001). Even when people were told that the solution implies to draw lines outside the given nine dots, most of the participants failed (Weisberg and Alba, 1981). Indeed, the majority of the naïve participants tried to solve the problem within the boundaries of the given nine dots. Öllinger et al. (2014) suggested that the Nine-Dot problem requires not only to relax the constraint, but also to have an appropriate strategy that helps to restrict the new and even larger search space, since drawing lines to non-dot points increases the search space exponentially.

**FIGURE 3:**
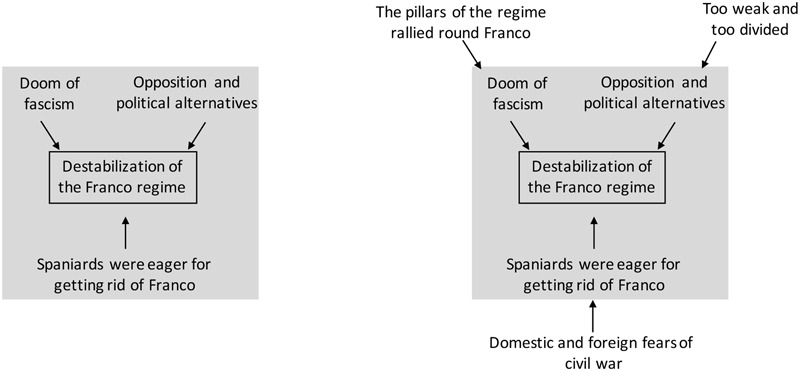
Left: Over-constrained problem representation. Arrows indicate a positive influence. Right: Actual problem representation.

In analogy to our historical problem constellation, it seems conceivable that political leaders selected strategies from a too narrow and constrained problem space (see **Figure 3**). The solution seemed obvious and was only a matter of time. The historical documents provide unequivocal evidence that political observers persisted on their assessments although the actual development was fairly different. Retrospectively, these misjudgements seem inexplicable, because most facts were already available from the beginning and were perceived accordingly. Considering these facts would have resulted in a more reliable expectation. This line of argumentation builds on the work of Klein (1999, 2008), who provided a model on natural decision making. Klein introduced a step-wise model which relies on a recognition-primed decision process. Initially, experts utilize familiar and already approved solution strategies. In more detail, incidents activate prior knowledge and decision makers evaluate, whether they already met such a situation. If so, they execute the according actions. Depending on how complicated the current incident is, further steps (mental simulations and evaluation) are necessary, until an appropriate solution strategy is selected.

Tetlock (1996, 1999) showed that providing counterfactual facts to experts about historical events leads to clearly biased problem representations. The representations were determined by the decision makers’ attitudes. The results revealed that experts neutralize dissonant data and preserve confidence in their prior assessments by resorting to a complex battery of belief-system defense. Tetlock showed that the results of what-if constructions are determined by the persons’ ideological world view. He argues that experts confronted with counterfactual evidences attempt to reduce the cognitive dissonance (Festinger, 1962) by ignoring or biasing the given evidences.

We suggest that our approach goes beyond a recognition account. We identified mental set as a driving force for ill-defined and biased problem representations that mainly drives the selection of decision making strategies.

## A Preliminary Model on Political Decision Making Based on Insight Problem Solving

In this section we outline a model on political decision making, which is based on cognitive processes stemming from the domain of insight problem solving.

**FIGURE 4:**
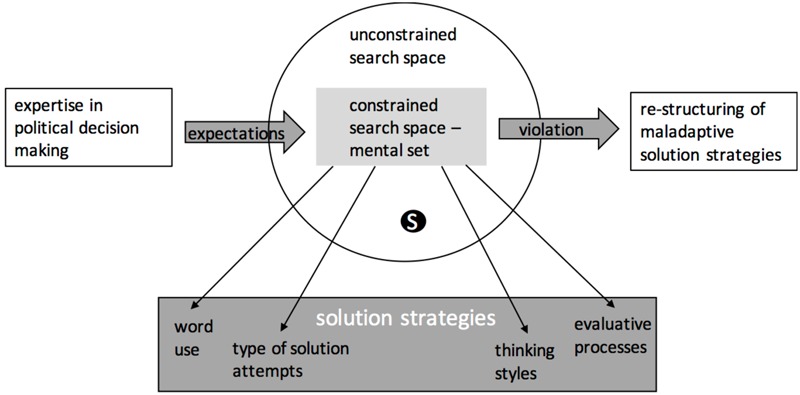
Model of expectation violation in political decision making. Expertise creates expectations, which result in a constrained search space. If the solution (S) lies outside the constrained search space, maladaptive solution strategies will occur. The realization of expectation violation is the key ingredient to change the solution strategy. Behaviorally, mental set manifests by the word use, the type of solution attempts, thinking styles, and evaluative processes.

As **Figure 4** depicts, experts in the field acquired a large corpus of domain related knowledge by their profession. However, this profound knowledge is affected by attitudes, interests, school of thoughts, prior experience, current political tendencies, the contemporary discourse, political systems, and general political opinions (Tetlock, 1996, 1999). The application of the biased knowledge to a new political situation or an unknown counterfactual scenario (Bar-Tal et al., 1989; Tetlock, 1999) may result in a mental set which over-constrains the search space. As a result, familiar and well-known, but insufficient strategies are applied. Unfortunately, those strategies will not necessarily solve the problem (Öllinger et al., 2014). We assume that political experts have high confidence in the reliability and validity of their knowledge. Consequently, they will probably repeatedly activate the maladaptive solution strategy and see no need to change the solution process. This behavior will induce a mental set. The mental set prevents the decision makers’ realization of expectation violation. Without the realization there is no drive to re-structure the self-imposed constrains. Realizing the violation of expectation will lead to a revised decision making process which relaxes the self-imposed constrains. As a result, the search space for a solution will be extended and a potential solution is accessible.

On a behavioral level, we assume that mental set is detectable by the used words (Cohn et al., 2004; Chung and Pennebaker, 2007; Pennebaker et al., 2015) (see our suggestions in the next section), the solution attempts, the thinking styles and the stream of the evaluative process.

## Testability of Our Assumption

To convey our case study and our model proposal into a testable research program, we derived the following hypotheses.

### Word Count Analysis of the Diplomatic Documentation

We aim at analyzing the large documentary evidence, on which our study is based, by means of quantitative methods. Pennebaker et al. (2001, 2003), Tausczik and Pennebaker (2010), and Pennebaker (2011) analyzed the frequency of words and made predictions about personality and thinking styles (e.g., function words or the frequency of used pronouns). We postulate that the documented assessments of Western politicians will show similar linguistic structures. This would demonstrate that an ideological mental set determines the search space for potential solutions to the discussed problems in Spain. According to Klein’s (2008) interpretation (see above), the situation was recognized as familiar and consequently, familiar solution strategies were applied.

### Field Studies

We plan to address the question how expertise and training in a particular political or historical domain (see Tetlock, 1999) will influence the decision making process. We are following Klein’s naturalistic decision making account (Klein, 1999, 2008) and plan to test experts from the field. We propose to confront them with fictive and complex historical and political scenarios, which the experts should evaluate. Then the experts will be asked to make predictions about the future developments of the scenarios. We hypothesize that the evaluation process will rather be determined by the specific expertise of the decision maker than by motivational factors like reducing cognitive dissonance (Tetlock, 1996).

### Laboratory Studies

Very recently, Salvi et al. (2016) demonstrated at a behavioral level that liberals solved problems significantly more often with insight than conservative people. The authors argue that both groups have different cognitive styles. In general, liberals are more flexible whereas conservatives are more rigid and prefer clear answers. Therefore, conservatives solve problems more analytically and in a step-by-step manner, whereas liberals solve the problems non-step-wise by insight.

Wiley (1998) showed that baseball experts, who were asked to use words from their domain knowledge in an unusually context, revealed a significant mental set. The mental set prevents the solution of problems, which refers to the domain of expertise, but was used in a more remote context.

Taking the evidences from both studies, we propose to test historical and political experts with problems that either require domain related knowledge or not. We suggest that expertise will inhibit innovative solutions, when decision makers are asked to find unusual solutions.

Moreover, it would be helpful to split the participants into two extreme groups. The criterion would be the participants’ cognitive styles. This would allow investigating the interaction between expertise and cognitive style and its impact on the solution of insight and non-insight problem solving as well as on political decision making.

## Conclusion and Perspectives

We demonstrated that insight problems violate the expectations of problem solvers by self-imposed constraints and mental set. We extrapolated these findings to more complex problems such as political decision making processes. By analyzing the considerations about Franco’s Spain we showed that in fact decision makers’ limited presuppositions biased the search space which consequently led to inappropriate expectations. Interestingly, the biased representation was not even updated although there was evidence which contradicted the initial expectations.

Given these findings, we conclude that expectation violation could be viewed as a general process that plays an eminent role even for complex problem solving. In our understanding both insight problem solving and political decision making could benefit from a better understanding and a more thorough and detailed analysis of expectations. A potential indication follows directly from this conclusion. We suggest that it might be worthwhile to monitor the problem solving process in order to detect the violation of expectation. The feedback of such a monitoring process might help the problem solvers to update, to elaborate or to restructure the search space and the related expectations.

Based on these findings we suggest further research in a twofold direction: on the one side, it would be helpful to enhance the described analogies between a determined historical setting and the psychological models by means of analyzing further unexpected historical developments, which led to the emergence of problems in view of a correct political perception of the events. This will provide the data base for attaining verifiable conclusions. On the other side, problem solving strategies in a political context should also be followed up in experimental studies. For example, it would be interesting to disentangle the different brain areas involved in the persistence of political expectations when the truth of these expectations is challenged by contradictory knowledge or experience.

## Author Contributions

MÖ contributed the psychological part. CCS provided the historiographical part. KM worked on the line of argumentation. AvM provided the philosophical and political background.

## Conflict of Interest Statement

The authors declare that the research was conducted in the absence of any commercial or financial relationships that could be construed as a potential conflict of interest.
